# Neutrophil-platelet ratio as a predictor of acute kidney injury in severe COVID-19

**DOI:** 10.1097/MD.0000000000040053

**Published:** 2024-10-11

**Authors:** Mihrican Sayan, Hatice Betul Altinisik, Ozan Sayan

**Affiliations:** a Department of Anesthesiology and Reanimation, Lapseki State Hospital, Canakkale, Turkey; b Department of Anesthesiology and Reanimation, Beykent University, Istanbul, Turkey; c Department of Anesthesiology and Reanimation, Canakkale Onsekiz Mart University, Canakkale, Turkey.

**Keywords:** acute kidney injury, blood cells, COVID-19, inflammation, intensive care

## Abstract

Acute kidney injury (AKI) is one of the most seen complications of coronavirus-2019 (COVID-19) infection. Patients with AKI caused by COVID-19 likely have higher neutrophil counts and lower platelet and lymphocyte levels. Therefore, the predictive value of many inflammation indexes calculated from the total blood count has been investigated to predict the AKI in COVID-19. According to our clinical experience, we thought that neutrophilia and thrombocytopenia may be more common in the development of AKI. For this reason, this study aimed to evaluate the predictive value of the neutrophil-to-platelet ratio (NPR) for AKI in severe COVID-19 patients. This retrospective study included 334 severe COVID-19 patients followed up in the intensive care unit (ICU). Predictive factors for AKI were analyzed. ROC curve analysis was performed to determine the inflammation indexes’ cutoff values for the AKI prediction. Multivariate analyses were performed to determine correlations between the inflammation indexes and AKI. In this study, AKI was determined at the rate of 43% (n:145). Independent risk factors affecting AKI were determined to be age (HR = 1.047, 95% confidence interval [CI]: 1.021–1.072, *P* < .001), the need for invasive mechanical ventilation (HR = 3.003, 95% CI: 1.645–5.481, *P* = .001) and the need for vasopressor (HR = 8.111, 95% CI: 3.786–17.375, *P < .001*). The optimal cutoff values predicting AKI were determined to be 3.9 for the NPR (AUC = 0.679, 95% CI: 0.622–0.737, *P* < .001) with 71.7% sensitivity and 61.9% specificity, 16.1 for the neutrophil-to-lymphocyte ratio (NLR) (AUC = 0.634, 95% CI: 0.575–0.694, *P* < .001) with 65.5% sensitivity and 56.1% specificity, and 3872.5 × 10^9^L for the systemic inflammatory index (SII) (AUC = 0.566, 95% CI: 0.504–0.629, *P* = .038) with 60% sensitivity and 55.6% specificity. In the regression model, only NPR values above the cutoff were related to AKI (HR = 3.817, 95% CI: 1.782–8.177, *P* = .001). The NPR has more predictive value than the NLPR, NLR, and SII in developing AKI in severe COVID-19 patients in the ICU. NPR is a new helpful index that can help clinicians predict early AKI in critical COVID-19.

## 1. Introduction

The first cases of coronavirus-2019 (COVID-19) infection caused by severe acute respiratory syndrome coronavirus-2 (SARS-COV-2) were determined in Wuhan, Hubei province, China, in December 2019. In a very short time, with rapid spread worldwide and high morbidity and mortality rates, COVID-19 was declared a global pandemic.^[1]^ Although pneumonia was the most severe symptom of COVID-19 infection, characterized by fever, cough, shortness of breath, and bilateral involvement in pulmonary imaging, the spectrum of COVID-19 ranged from asymptomatic to fatal.^[2]^ There are several comorbidities and underlying reasons for comorbidities causing the progression of COVID-19 to severe disease (defined as requiring hospitalization, intensive care, intubation, mechanical ventilation, or death).

Acute kidney injury (AKI) is one of the most seen complications of COVID-19 infection. The frequency of AKI at the time of first hospital presentation has been reported to be 17%, and this rate increases to 45% in severe COVID-19 patients who require intensive care unit (ICU) follow-up.^[3]^ Although the definitive mechanism underlying COVID-19-related AKI is not fully known, there has been a focus on many mechanisms, such as hemodynamic instability, direct cytotoxic damage of the virus, systemic abnormal immune response, and damage mediated by ACE-2 receptors.^[4]^ AKI has been shown to increase mortality in these patients.^[5]^ Therefore, early detection of AKI and aggressive treatment are crucial for survival. The predictive value of many biomarkers has been investigated to be able to predict the severity of COVID-19. To date, it has been suggested that laboratory values such as lymphocyte count, neutrophil-to-lymphocyte ratio (NLR), thrombocyte-lymphocyte ratio (PLR), C-reactive protein (CRP), ferritin, troponin, D-dimer could be proper in the evaluation of the risk of mortality and critical disease.^[6,7]^ This has led to the search for a test that could show a specific prognosis of multisystem involvement in COVID-19. The most recent studies of the sensitivity and specificity of the systemic inflammatory index (SII) and neutrophil to lymphocyte*platelet ratio (NLPR) have been conducted to predict COVID-19 mortality.^[8,9]^

Patients with AKI caused by COVID-19 had higher neutrophil counts and decreased platelet and lymphocyte levels.^[10]^ Furthermore, these individuals have higher comorbidities and require intensive care. Although the relationship between COVID-19, platelet count changes, and kidney health is known, the detailed mechanisms are still ongoing research topics. Our clinical findings show that decreased platelet count and elevated neutrophil count were observed more frequently in patients with AKI. As a result, we reasoned that the neutrophil-to-platelet ratio (NPR) could be a more effective test for predicting kidney injury in COVID-19. Therefore, this study aimed to evaluate the predictive value of the NPR for AKI in severe COVID-19 patients.

## 2. Methods

### 2.1. Study design and setting

The study used a retrospective cross-sectional design, and the Strengthening the Reporting of Observational Studies in Epidemiology (STROBE) guideline was followed. Investigation was made of patients treated for COVID-19 pneumonia in the Anesthesiology and Reanimation ICU between March 15, 2020, and March 15, 2022. Patient data were retrieved from the hospital information system and recorded anonymously.

## 3. Ethics

Approval for this study was granted by the Clinical Research Ethics Committee (Chairman: Prof C. Silan) of Çanakkale University (project no: 2023-80, decision no: 2023/09-07, dated: June 21, 2023). All procedures complied with the principles of the Helsinki Declaration. The requirement for informed consent was waived to protect the anonymity of the data.

## 4. Participants

A total of 1001 patients were treated for COVID-19 in the ICU between relevant dates and met the criteria for inclusion.

The inclusion criteria were

- Patients with severe COVID-19 and a positive polymerase chain reaction test (PCR)
- Patients with available blood cell counts and serum creatinine (SCr) at baseline
- Patients with complete demographic data, laboratory data, and therapeutic interventions

The exclusion criteria were

- Patients with age under 18
- Patients with suspected COVID-19 and a negative PCR test
- Patients with AKI on the day of ICU admission
- Patients with non-severe COVID-19
- Patients with chronic renal disease, cancer, and blood cell proliferation

**Figure 1.**
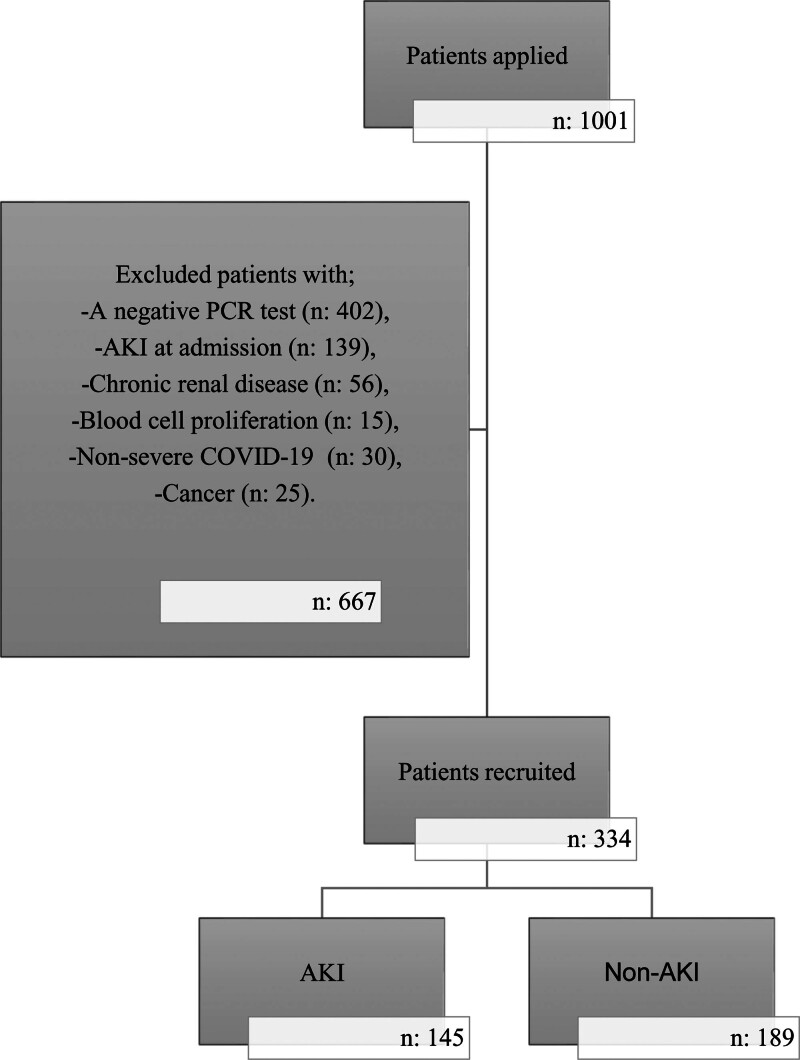
Study flow diagram.

Finally, 334 patients were included in the present study (Fig. 1).

## 5. Data collection and definitions

A record was made for each patient of the demographic data, treatments received for COVID-19, survival status, requirement for invasive mechanical ventilation, vasopressor, oxygen fraction in inspired gas mixture (FiO_2_) values, and length of stay in ICU from the hospital information system.

Clinical laboratory technicians performed the biochemical tests in the hospital-certified laboratory, and the values of urea, SCr, partial oxygen pressure in arterial blood (PaO_2_), hemogram, procalcitonin, and CRP were recorded.

The PaO_2_/FiO_2_ ratio, NLR (neutrophil/lymphocyte), NPR (neutrophil*100/platelet), NLPR (neutrophil*100/lymphocyte*platelet), and SII (platelet*neutrophil/lymphocyte) were calculated from the recorded values.

Patients with AKI were identified according to the Kidney Disease Improving Global Outcomes (KDIGO) criteria. Two groups were formed of patients with AKI (n = 145) or without AKI (n = 189). According to KDIGO criteria, patients with a ≥50% increase in SCr within 7 days or a ≥0.3-mg/dL (≥26.5-μmol/L) rise in SCr within 48 hours or oliguria were defined as patients with AKI (n = 145).^[11]^

## 6. Statistical analysis

The analyses were performed between non-AKI and AKI groups. Missing data excluded. Data obtained in the study were analyzed statistically using IBM SPSS vn. 26.0 software. Conformity of the data to normal distribution was assessed using the Shapiro–Wilk test. nonparametric tests were used in the analyses as the data did not show normal distribution. Accordingly, comparisons between the 2 groups were performed with the Mann–Whitney U-test. The Pearson Chi-square test was used to examine the relationships between categorical variables. The ability of the laboratory values to predict AKI was evaluated with ROC analysis. According to steroid use, an additional ROC analysis was performed for neutrophil-based predictive measures (neutrophil count, NLR, NLPR, NPR, SII). After roc analysis, optimal cutoff values were determined using the Youden index. Prediction values were divided into 2 groups according to cutoff values (below and above the cutoff point). For neutrophil-based predictive measures, patients were grouped as above or below the cutoff values determined for those who received steroids and those who did not. Variables that were statistically significant for AKI in the univariate analyses (age, hypertension, need for invasive mechanical ventilation, need for vasopressor) and variables grouped according to cutoff values (PaO_2_/ FiO_2_, NLR, SII, NLPR, NPR, neutrophil, lymphocyte, platelet) were included in the logistic regression analysis. Path analysis was performed with the SPSS Amos 21 program. The statistically significant results of numerical variables not showing normal distribution were reported as median and interquartile range (IQR, 25th–75th percentile) values and categorical variables as number (n) and percentage (%). Results were presented in a 95% confidence interval (CI) at a statistical significance of *P* = .05.

## 7. Results

The study included 334 patients who were followed up in ICU because of COVID-19 infection. The patients comprised 135 (40%) females and 199 (60%) males with a median age of 69 years (IQR, 25th–75th, 59–76), with comorbidities of diabetes mellitus determined in 137 (41%), hypertension (HT) in 171 (51%), and cardiovascular system disease in 96 (29%). The mortality rate was determined to be 57% (survivors n = 144, non-survivors n = 190). The length of stay in the ICU was a median of 12 days and 205 (61%) patients required invasive mechanical ventilation. The frequency of the development of AKI in COVID-19 patients followed up in ICU was determined to be 43% (n = 145).

### 7.1. Results related to acute kidney injury

The patients with AKI were determined to be older with a higher frequency of HT than patients who did not develop AKI. The rate of need for invasive mechanical ventilation and the need for vasopressors were statistically significantly higher in the patients with AKI than in the patients without AKI. No statistically significant association was found between treatments used for COVID-19 and the development of AKI (Table 1).

**Table 1:** Analyses of the demographic, clinical, and laboratory data according to the development of acute kidney injury.

(n, %), (median, IQR 25th–75th)	Total (n = 334)	Non-AKI (n = 189)	AKI (n = 145)	*P*-value
Age (yr)	69 (59–76)	65 (55–73)	73 (65–80)	<.001
Gender (F/M)	135 (40%) vs 199 (60%34	76 (40%) vs 113 (60%)	59 (41%) vs 86 (59%)	.930
Mortality (present/absent)	190 (57%) vs 144 (43%)	73 (39%) vs 116 (61%)	117 (81%) vs 28 (19%)	<.001
Need for vasopressor (present/absent)	62 (19%) vs 272 (81%)	17 (9%) vs 172 (91%)	45 (31%) vs 100 (69%)	<.001
DM (present/absent)	137 (41%) vs 197 (59%)	71 (38%) vs 118 (62%)	66 (46%) vs 79 (54%)	.143
HT (present/absent)	171 (51%) vs 163 (49%)	81 (43%) vs 108 (57%)	90 (62%) vs 55 (38%)	<.001
CVSD (present/absent)*	96 (29%) vs 238 (71%)	49 (26%) vs 140 (74%)	47 (32%) vs 98 (68%)	.194
ICU length of stay (d)	11 (7–17)	11 (7–17)	12 (7–16)	.623
Invasive mechanical ventilation (present/absent)	205 (61%) vs 129 (39%)	94 (50%) vs 95 (50%)	111 (77%) vs 34 (23%)	<.001
Administration of favipiravir (present/absent)	268 (80%) vs 66 (20%)	150 (79%) vs 39 (21%)	118 (81%) vs 27 (19%)	.647
Administration of tocilizumab (present/absent)	48 (14%) vs 286 (86%)	31 (16%) vs 158 (84%)	17 (12%) vs 128 (88%)	.227
Administration of anakinra (present/absent)	10 (3%) vs 324 (97%)	3 (2%) vs186 (98%)	7 (5%) vs138 (95%)	.109
Administration of plaquenil (present/absent)	24 (7%) vs 310 (93%)	17 (9%) vs 172 (91%)	7 (5%) vs 138 (95%)	.144
Administration of colchicine (present/absent)	40 (12%) vs 29 (88%)	24 (13%) vs 165 (87%)	16 (11%) vs 129 (89%)	.642
Administration of steroid†				
Absent	63 (19 %)	36 (19%)	27 (19%)	.907
Low dose	151 (45%)	87 (46%)	64 (44%)	
High dose	120 (36%)	66 (35%)	54 (37 %)	
Administration of convalescent plasma (present/absent)	132 (40%) vs 202 (61%)	81 (43%) vs 108 (57%)	51 (39%) vs 94 (65%)	.154
PaO_2_/FiO_2_	89 (69–137)	92 (74–147)	83 (66–127)	.009
CRP (mg/L)	9.2 (5.0–16.0)	9.3 (5.0–15.1)	9.2 (5.3–17.0)	.276
Procalcitonin (ng/mL)	0.27 (0.12–0.75)	0.21 (0.11–0.58)	0.32 (0.14–1.06)	.007
Neutrophils (×10^9^/L)	9.7 (7–13.3)	9.0 (6.8–12.1)	11.0 (7.8–15.0)	.001
Lymphocytes (×10^9^/L)	0.58 (0.37–0.80)	0.60 (0.40–0.88)	0.50 (0.30–0.77)	.004
Thrombocytes (×10^9^/L)	242 (183–301)	252 (195–330)	218 (160–284)	.002
NLR	16.9 (10.3–26.3)	14.7 (9.2–22.5)	21.0 (12.6–34.1)	<.001
SII (×10^9^/L)	4004 (2242–6950)	3580 (2116–6364)	4585 (2379–7959)	.038
NLPR	7 (4–12)	6 (3–10)	10 (6–16)	<.001
NPR	4 (3–6)	3 (2–5)	5 (4–7)	<.001

All patients received low molecular weight heparin therapy, with dosage adjustments based on the glomerular filtration rate.

AKI = acute kidney injury, CRP = C-reactive protein, CVSD = cardiovascular system disease, DM = diabetes mellitus, F = female, HT = hypertension, ICU = intensive care unit, M = male, NLPR = neutrophil–lymphocyte*platelet ratio, NLR = neutrophil–lymphocyte ratio, NPR = neutrophil–platelet ratio, PaO2/FiO2 = partial oxygen pressure in arterial blood to the oxygen fraction in inspired gas mixture, PCT = procalcitonin, SII = systemic inflammatory index.

* Includes coronary artery disease, heart valve disease, and heart failure.

† Low dose: less than 250 mg of methylprednisolone; high dose: 250 mg or more.

In the examination of the laboratory parameters measured on admission to ICU, the values of the patients with AKI were determined to be higher than those of the patients without AKI in respect of procalcitonin, NLR, SII, NLPR, NPR and were lower in respect of PaO_2_/FiO_2_. No significant difference was observed between the patients with and without AKI regarding the CRP values (Table 1).

**Figure 2.**
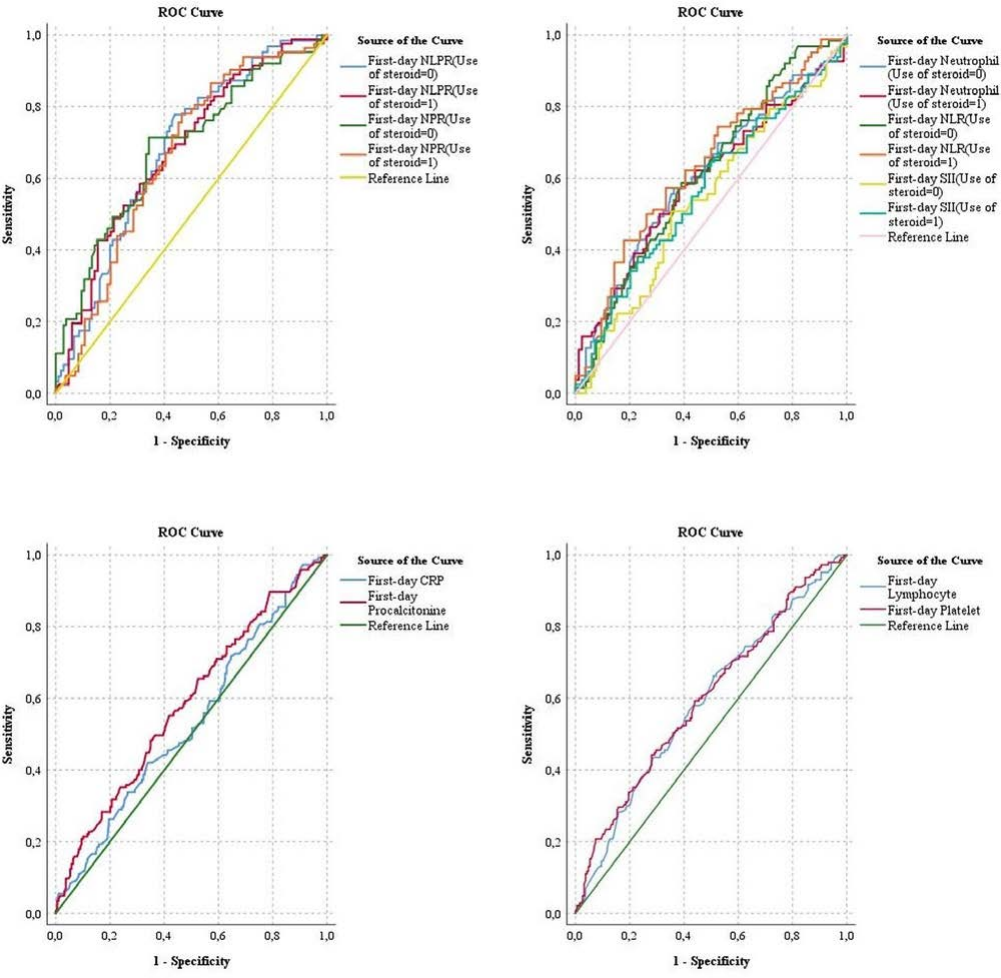
ROC curves of inflammatory markers. CRP: C-reactive protein; NLR: neutrophil-to-lymphocyte ratio; SII: systemic inflammatory index; NLPR: neutrophil-to-lymphocyte*platelet ratio; NPR: neutrophil-to-platelet ratio.

In the ROC analyses of the inflammatory markers in the prediction of AKI (Table 2), the optimal cutoff values were determined to be 3.9 the NPR (AUC = 0.679, 95% CI: 0.622–0.737, *P* < .001) with 71.7% sensitivity and 61.9% specificity, 16.095 for the NLR (AUC = 0.634, 95% CI: 0.575–0.694, *P* < .001) with 65.5% sensitivity and 56.1% specificity, and 3872.5 × 10^9^L for the systemic SII (AUC = 0.566, 95% CI: 0.504–0.629, *P* = .038) with 60% sensitivity and 55.6% specificity. On the day of admission to the ICU, patients who received steroids in the hospital had higher neutrophil counts (median IQR: 10.7 [7.9–14.9] × 10^9^/L vs 8.8 [6.5–12.1] × 10^9^/L, *P* < .001) (Table 3). A second ROC analysis was conducted for neutrophil measures based on steroid use. cutoff values for patients who received and did not receive steroids were presented. In the ROC analyses based on steroid use, the SII predictive value was not statistically significant. However, other neutrophil-containing values significantly predicted AKI (Table 2). ROC curves are shown in Figure 2. Furthermore, the association of these markers with steroid and vasopressor treatments, in addition to invasive mechanical ventilation, was investigated in patients who developed AKI. There were no significant differences in NPR values between any groups (Table 3).

**Table 2:** Prognostic values of inflammation markers and blood cell counts in predicting acute kidney injury, ROC analysis.

Test	Use of Steroid	AUC	95% CI	Youden index	Cutoff	*P*	Sensitivity (%)	Specificity (%)	Positive predictive value (%)
First-day NLR	Total	0.634	0.575 to 0.694	0.216	>16.095	<.001	65.5	56.1	53
	Absent	0.612	0.525 to 0.698	0.197	>14.98	.011	58.7	61.0	48
	Present	0.635	0.551 to 0.720	0.248	>27.99	.002	42.7	82.1	70
First-day SII	Total	0.566	0.504 to 0.629	0.156	>3872.5	.038	60	55.6	50
	Absent	0.541	0.450 to 0.632	0.156	>3845.2	.374	50.8	64.8	38
	Present	0.568	0.480 to 0.655	0.158	>4866.8	.130	63.4	52.4	57
First-day NLPR	Total	0.685	0.628 to 0.742	0.295	>5.4	<.001	80.7	49.2	55
	Absent	0.680	0.599 to 0.761	0.340	>5.4	<.001	77.8	56.2	52
	Present	0.679	0.598 to 0.760	0.274	>11.3	<.001	52.4	75	67
First-day NPR	Total	0.679	0.622 to 0.737	0.336	>3.9	<.001	71.7	61.9	59
	Absent	0.685	0.601 to 0.769	0.371	>3.9	<.001	71.4	65.7	55
	Present	0.661	0.577 to 0.745	0.316	>3.7	<.001	78.0	53.6	62
First-day neutrophil	Total	0.608	0.546 to 0.670	0.200	>10.92	.001	51.7	68.3	56
	Absent	0.606	0.516 to 0.696	0.210	>9.65	.021	57.1	63.8	49
	Present	0.596	0.509 to 0.683	0.192	>10.92	.031	57.3	61.9	60
First-day procalcitonine	Total	0.585	0.524 to 0.647	0.134	>0.296	.006	55.2	58.2	50
CRP	Total	0.535	0.472 to 0.597	0.082	>11.9	.137	42.1	66.1	49
First-day lymphocyte	Total	0.591	0.539 to 0.662	0.154	≤0.615	.004	66.2	49.2	50
First-day platelet	Total	0.601	0.539-0.662	0.161	≤204.5	.001	44.1	72	55

AKI = acute kidney injury, AUC = area under the ROC curve, CI = confidence interval, CRP = C-reactive protein, NLPR = neutrophil-lymphocyte*platelet ratio, NLR = neutrophil-lymphocyte ratio, NPR = neutrophil- platelet ratio, PCT = procalcitonin, SII = systemic inflammatory index.

**Table 3:** The association between IMV, steroid and vasopressor use, and biomarkers in patients with AKI

(Median, IQR 25th–75th)	IMV in AKI patients			Use of vasopressors in AKI patients*			Use of steroids in AKI patients			Use of steroids in all patients†		
	Present (n = 34)	Absent (n = 111)	*P*-value	Present (n = 33)	Absent (n = 112)	P-value	Present (n = 82)	Absent (n = 63)	*P*-value	Present (n = 166)	Absent (n = 168)	*P*-value
First-day neutrophil (×10^9^/L)	11.1 (8.0–14.9)	9.4 (5.9–16.5)	.285	12.3 (7.8–14.4)	10.9 (7.7–15.6)	.784	11.9 (8.1–16.5)	10.5 (7.3–13.3)	.064	10.7 (7.8–14.9)	8.8 (6.5–12.1)	.001
First-day lymphocyte (×10^9^/L)	0.50 (0.30–0.70)	0.55 (0.30–1.00)	.296	0.41 (0.25–0.60)	0.52 (0.30–0.80)	.148	0.41 (0.27–0.67)	0.60 (0.40–0.80)	.005	0.50 (0.30–0.87)	0.60 (0.40–0.78)	.001
First-day platelet (×10^9^/L)	227 (160–284)	210 (171–280)	.913	211 (147–253)	223 (162–292)	.279	241 (171–312)	204 (157–254)	.126	250 (184–331)	238 (183–284)	.240
First-day NLPR (×10^9^/L)	10.5 (6.3–16.3)	6.5 (4.8–13.8)	.023	12.3 (6.7–27.0)	9.5 (5.8–14.2)	.067	11.4 (6.7–18.4)	7.9 (5.5–12.0)	.008	9.3 (4.8–14.5)	5.9 (3.8–10.7)	<.001
First-day NLR	21.9 (13.5–34.3)	16.5 (8.8–29.0)	.046	24.0 (14.0–46.1)	20.6 (12.7–30.7)	.242	24.5 (15.6–41.7)	16.5 (11.2–24.4)	.001	21.4 (12.9–31.7)	14.1 (8.6–21.4)	<.001
First-day NPR	4.9 (3.9–7.6)	4.1 (3.0–6.2)	.101	5.2 (3.9–7.4)	4.6 (3.5–6.5)	.363	4.8 (3.7–7.1)	4.7 (3.2–6.5)	.786	4.1 (2.6–5.5)	3.9 (2.6–5.5)	.067
First-day SII (×10^9^/L)	4910 (–7958)	3365 (1984–7656)	.119	4656 (2641–7875)	4461 (2337–7849)	.664	5642 (2911–10,694)	3885 (2045–5005)	.002	5270 (2680–8042)	3342 (1842–5365)	<.001

AKI = acute kidney injury, IMV = invasive mechanical ventilation, NLPR = neutrophil-lymphocyte*platelet ratio, NLR = neutrophil-lymphocyte ratio, NPR = neutrophil-platelet ratio, SII = systemic inflammatory index.

* This group includes the use of any vasopressor agents before the development of AKI in patients who eventually developed AKI.

† Patients were grouped based on whether they received steroids during their hospital stay before admission to the intensive care unit.

Logistic regression analysis was performed on the model created from the factors determined to have a statistically significant effect on the development of AKI in the univariate analyses and variables grouped according to cutoff values (PaO_2_/FiO_2_, NLR, SII, NLPR, NPR, neutrophil, lymphocyte, and platelet) (*P* = .016, Nagelkerke *R* Square = 0.432). According to the results, age, the need for invasive mechanical ventilation, and vasopressor were determined to be independent risk factors causing AKI. Furthermore, NPR above the cutoff value and PaO_2_/FiO_2_ levels below the cutoff value were independent risk factors for AKI (Table 4).

**Table 4:** Logistic regression analysis for acute kidney injury after grouping the predictive values according to the cutoff values - (*P* = .016, Nagelkerke *R* Square = 0.432).

Independent variables	β	Exp (β)	95% confidence interval		*P*-value
			Lower limit	Upper limit	
Age	0.046	1.047	1.021	1.074	<.001
Hypertension	0.453	1.573	0.906	2.731	.107
Need for invasive mechanical ventilation	1.100	3.003	1.645	5.481	<.001
Need for vasopressor	2.093	8.111	3.786	17.375	<.001
First-day PaO_2_/FiO_2_*	1.382	3.984	2.102	7.549	<.001
First-day procalcitonin†	-0.364	0.695	0.387	1.245	.221
First-day neutrophil†	-0.038	0.963	0.463	2.005	.920
First-day NLR†	0.165	0.679	0.539	2.102	.679
First-day NLPR†	-0.228	0.796	0.352	1.798	.583
First-day NPR†	1.339	3.817	1.782	8.177	.001
First-day platelet*	0.429	1.535	0.786	2.998	.210
First-day lymphocyte*	0.147	1.160	0.586	2.295	.670

Logistic regression analysis.

AKI = acute kidney injury, NLPR = neutrophil-lymphocyte*platelet ratio, NLR = neutrophil-lymphocyte ratio, NPR = neutrophil- platelet ratio, PaO2/FiO2 = partial oxygen pressure in arterial blood to the oxygen fraction in the inspired gas mixture, SII = systemic inflammatory index.

* Smaller values are associated with AKI. cutoff values; PaO_2_/FiO_2_ = 69.5, platelet = 204.5, lymphocyte = 0.615.

† Larger values are associated with AKI. Variables other than procalcitonin were grouped according to their cutoff values determined by steroid use. The cutoff values are as follows: procalcitonin = 0.296; neutrophil count with steroids = 10.92, without steroids = 9.65; NLR with steroids = 27.99, without steroids = 14.98; NLPR with steroids = 11.3, without steroids = 5.4; NPR with steroids = 3.7, without steroids = 3.9.

**Figure 3.**
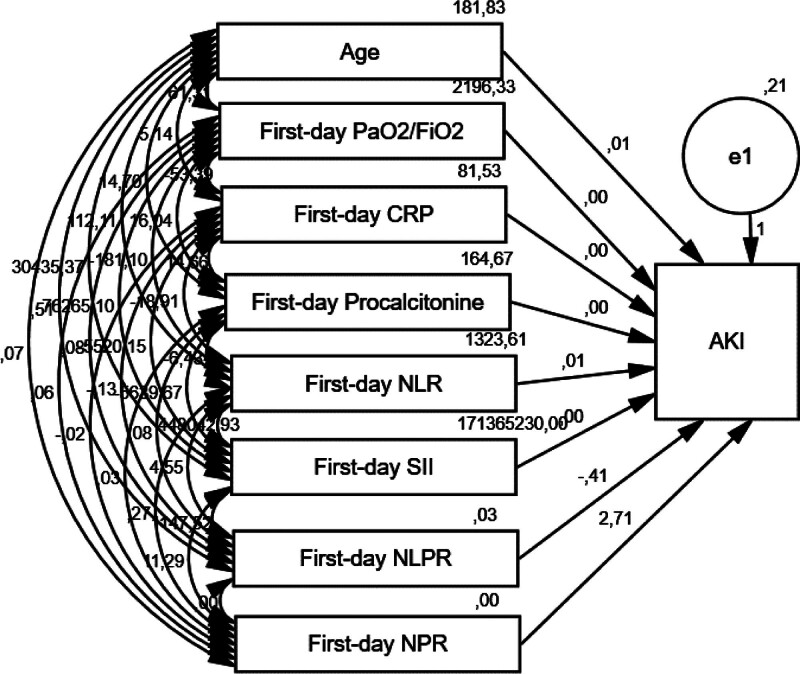
Path analyses of markers. AKI: acute kidney injury, CRP: C-reactive protein, NLR: neutrophil-to-lymphocyte ratio, SII: systemic inflammatory index, NLPR: neutrophil-to-lymphocyte*platelet ratio, NPR: neutrophil-to-platelet ratio.

We hypothesized that the direct effect of NPR on AKI incidence would be more potent compared to other single inflammatory indicators. Path analysis was performed to examine the association of different inflammatory indicators with AKI (Fig. 3 and Table 5). The results showed that NPR had the strongest positive association with the incidence of AKI (β = 2.705, *P* = .006) compared with NLR (β = 0.005, *P* = .197) and SII (β < 0.001, *P* = .286).

**Table 5:** Path analyses for acute kidney injury.

Hypothesis	Estimate	Standard Error	*P*-value	Support
Age → AKI	0.010	0.002	<.001	Yes
PaO_2_/FiO_2_ → AKI	−0.001	0.001	.012	Yes
CRP → AKI	0.003	0.003	.345	No
Procalcitonine → AKI	0.002	0.002	.245	No
NLR → AKI	0.005	0.004	.197	No
SII→ AKI	<0.001	<0.001	.286	No
NLPR → AKI	−0.412	0.365	.259	No
NPR→ AKI	2.705	0.991	.006	Yes

AKI = acute kidney injury, NLPR = neutrophil-lymphocyte*platelet ratio, NLR = neutrophil-lymphocyte ratio, NPR = neutrophil- platelet ratio, PaO_2_/FiO_2_ = partial oxygen pressure in arterial blood to the oxygen fraction in the inspired gas mixture, SII = systemic inflammatory index.

## 8. Discussion

This cohort study aimed to examine the risk factors related to the development of AKI in 334 patients with COVID-19 infection in the ICU. The study results demonstrated that age, PaO_2_/FiO_2_, NPR, the need for vasopressor, and invasive ventilation were independent predictors of AKI in critical COVID-19 patients in the ICU. The crucial predictive value of NPR for AKI risk was determined by ROC analysis. More importantly, as compared to NLR, SII, NLPR, CRP, and procalcitonin, NPR may be the most effective inflammatory indicator to predict the probability of AKI in severe COVID-19 patients.

The underlying cause of kidney damage caused by COVID-19 is complicated. The primary reasons include prerenal azotemia, acute tubular injury, glomerulopathy, thrombotic microangiopathy, and complications of COVID-19 treatment.^[10,12]^ Many of these factors are linked to the hyperinflammatory response. Cytokines induce renal endothelial cells and play a role in microcirculation dysfunction by causing renal vascular permeability.^[13]^ In addition, cell death and tissue damage can occur associated with high cytokine levels in circulation, leading to kidney organ failure.^[14]^ As a result of pro-inflammatory cytokines, deep T-cell lymphopenia and reduced numbers of dendritic cells directed by T-cell sequestration in tissues or T-cell apoptosis are common in severe patients.^[15]^ It leads to an imbalance in innate and acquired immune responses, neutrophils and macrophage hyperactivation, and a delay in the clearance of viruses.^[16]^ Complement activation and procoagulation pathways can stimulate each other. In most patients with severe COVID-19, clotting activity increases, and as a result, the consumption of clotting factors leads to microvascular thrombosis.^[17]^ Thrombocytopenia is common in critically ill patients and mainly demonstrates crucial organ dysfunction or physiological decompensation rather than primary hematological reasons. It is frequently related to the development of disseminated intravascular coagulation.^[18]^ Disease severity, organ dysfunction, sepsis, vasopressor treatment, and renal failure are all risk factors for thrombocytopenia acquired in the ICU.^[19]^ The mechanism of thrombocytopenia in COVID-19 patients is most likely complex in nature, with decreased production, increased consumption, and destruction varied according to disease severity, multiple organ failure, and shock.^[18,20]^ Although the underlying cause of thrombocytopenia is not fully understood, coagulopathy and thrombosis are also particularly important in COVID-19. These modifications in blood cells led researchers to investigate their relationship with kidney injury.

Many studies have shown SII, NLR, and NLPR as predictive and prognostic indicators for kidney injury. These indices result from the formulation that uses platelet and neutrophil-lymphocyte counts. In a study of hospitalized COVID-19 patients, Cheng et al showed that thrombocyte and lymphocyte counts were lower in those who developed AKI, and leukocyte counts were higher.^[5]^ In consecutive analyses, neutrophilia, thrombocytopenia, and lymphopenia were present in patients with AKI. These results appear to be consistent with the literature. In a study examining 399 COVID-19 patients in an (ICU), Samra et al reported that patients with thrombocytopenia had a higher risk of developing AKI. Additionally, they identified the platelet cutoff value as a risk factor for kidney injury to be 152.5 (AUC = 0.75).^[21]^ Similarly, in univariate analyses of our study, low platelet count was associated with an increased risk of developing AKI; however, the ROC analysis cutoff value was higher (cutoff value: 204.5, AUC: 0.601).

The results performed on NLR have produced conflicting findings. An elevated NLR was reported to be an independent predictor for the development of AKI in hospitalized COVID-19 patients.^[22]^ Chen et al determined a correlation between AKI progression and mortality in patients with low-grade AKI and NLR values <7 and >38.^[23]^ On the other hand, Bravo et al reported that the NLR did not show a significant difference between COVID-19 patients with and without kidney injury.^[9]^ In the current study, the NLR values were higher in the COVID-19 patients in ICU with AKI. In the ROC analyses performed to determine the predictive value of NLR for the development of AKI, the cutoff values were found to be significant at 16.1 for NLR (AUC = 0.634, *P* < .001).

There is insufficient information regarding the role of SII in predicting the development of AKI in COVID-19. An SII cutoff value of 1145 for the prediction of ICU requirement (AUC = 0.752) and mortality (AUC = 0.714) in 177 hemodialysis patients with COVID-19 determined by Sevinç et al.^[24]^ However, there is no study on the predictive value of SII in the development of AKI in COVID-19. In the present study, the SII values were correlated with the onset of AKI in patients with severe COVID-19. The optimal threshold determined for this index has shown statistical significance for estimating the AKI (cutoff = 3872.5, AUC = 0.566, *P* = .038).

Recent studies have investigated the predictive value of NLPR in the context of COVID-19. According to Bravo et al, NLPR is superior to NLR in predicting the risk of AKI in COVID-19 patients. It has been shown that a threshold of 5 (AUC = 0.670) is significant in predicting AKI.^[9]^ Contreras-Chavez et al focused on the course of AKI in individuals with sepsis caused by COVID-19 infection.^[25]^ Their findings revealed that a significant proportion (68.4%) of patients with NLPR higher than 3 were observed with AKI. The current study found a cutoff value of 5.4 (AUC = 0.685, *P* < .001) in predicting AKI development in patients with severe COVID-19.

Our study found that the NPR is a more valuable predictor of the risk of AKI compared to other indices. The NPR cutoff value found in our study was 3.9 (AUC = 0.679, *P* < .001), while in patients who received steroids, the cutoff value was 3.7 (AUC = 0.661, *P* < .001). The NPR showed the most potent direct effect in the pathway analysis to assess the strength of biomarkers. In multivariate analyses, values above this cutoff threshold were associated with a 3.8-fold increase in the risk of developing AKI in severe COVID-19 patients. Additionally, in patients who developed AKI, the NPR value did not show a significant difference based on steroid use, vasopressor use, or invasive mechanical ventilation. Although the development of AKI in severe COVID-19 patients is multifactorial, NPR values demonstrated a more specific relationship compared to other indices. To date, no study has been conducted in the literature that addresses the NPR ratio in the context of COVID-19. In this context, our work offers crucial contributions to literature.

## 9. Limitations

This study was a single-center, retrospective, and observational research, and the inherent bias was unavoidable. There is a need for more high-quality studies on this issue. Unfortunately, the AUC values in our study do not constitute a gold standard test; therefore, these findings need to be corroborated with clinical evaluation and clinician support.

## 10. Conclusion

Consequently, in this cohort analysis, the NPR was the most important predictor of AKI in severe COVID-19 patients in the ICU. NPR can assist in clinical decision-making and patient treatment. With the lack of literature addressing the significance of NPR in COVID-19, our findings provide a significant contribution and serve as a guide for future studies and possible clinical applications. As a result, our work highlights the complex nature of COVID-19 pathophysiology and the critical relevance of novel biomarkers in predicting the outcomes of individuals most at risk for severe consequences.

## Author contributions

**Conceptualization:** Mihrican Sayan, Hatice Betul Altinisik, Ozan Sayan.

**Data curation:** Ozan Sayan.

**Formal analysis:** Mihrican Sayan.

**Investigation:** Mihrican Sayan, Hatice Betul Altinisik, Ozan Sayan.

**Methodology:** Mihrican Sayan, Hatice Betul Altinisik, Ozan Sayan.

**Resources:** Mihrican Sayan.

**Supervision:** Hatice Betul Altinisik.

**Validation:** Mihrican Sayan.

**Visualization:** Mihrican Sayan.

**Writing – original draft:** Mihrican Sayan, Ozan Sayan.

**Writing – review & editing:** Hatice Betul Altinisik, Ozan Sayan.
